# First person – Geon Seong Lee

**DOI:** 10.1242/dmm.049312

**Published:** 2021-10-25

## Abstract

First Person is a series of interviews with the first authors of a selection of papers published in Disease Models & Mechanisms, helping early-career researchers promote themselves alongside their papers. Geon Seong Lee is first author on ‘ *Morc2a* p.S87L mutant mice develop peripheral and central neuropathies associated with neuronal DNA damage and apoptosis’, published in DMM. Geon Seong is a PhD student in the lab of Su Cheong Yeom at Seoul National University, Pyeongchang, South Korea, investigating gene-editing therapy for single-nucleotide polymorphism-mediated genetic disorder.


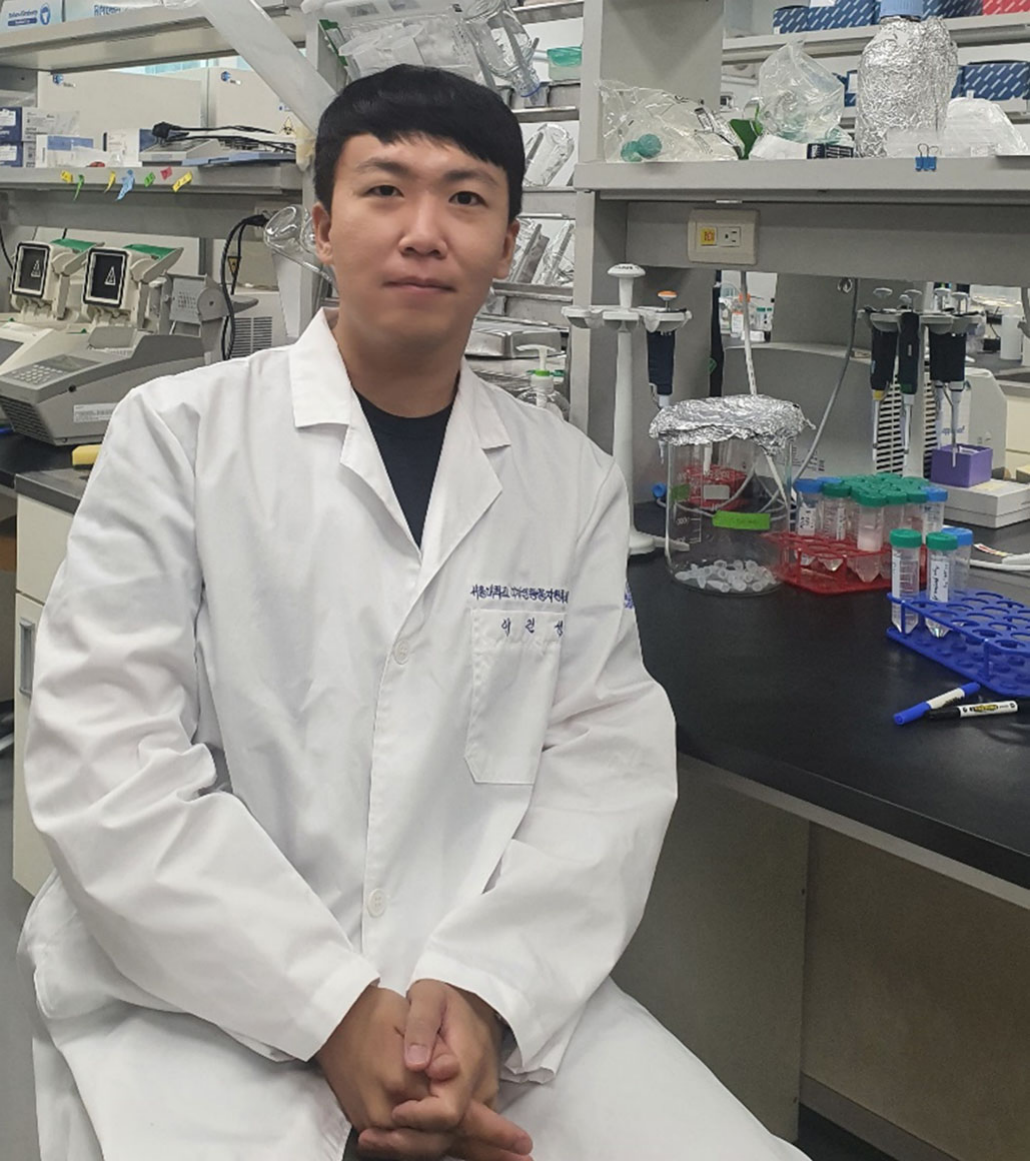


**Geon Seong Lee**

**How would you explain the main findings of your paper to non-scientific family and friends?**

Charcot-Marie-Tooth disease (CMT) is a typical genetic disease known as peripheral neuropathy. *MORC2* missense mutation is known to cause CMT type 2 Z (CMT2Z) of peripheral axonal neuropathy. However, it has been reported that some patients develop not only peripheral nerves but also central neuropathy. Since the aetiology of MORC2-mediated neuropathy is uncertain, we used CRISPR/Cas9 to generate one of the *MORC2* variants, the S87L mutant mouse. These mice exhibited central neuropathy with neuronal degeneration as well as the clinical symptoms of human CMT2Z patients. Our novel animal is expected to be a model with strong potential to study *MORC2*-induced neuropathies.

**What are the potential implications of these results for your field of research?**

*MORC2*-mediated neuropathy was first discovered in 2016. Various *MORC2* variants have been found in patients with the disease, but the exact cause and mechanism are not yet known. In our study, S87L mutant mice were first generated using CRISPR/Cas9. Our *Morc2a* S87L/+ mice have revealed an unknown cause of disease in patients, and these mice have potential use in disease therapy studies.

**What are the main advantages and drawbacks of the model system you have used as it relates to the disease you are investigating?**

Our *Morc2a* S87L/+ mice are the first *MORC2* mutation disease models. Because CMT2Z and developmental delay, impaired growth, dysmorphic facies and axonal neuropathy (DIGFAN) diseases still have many unknown points, the animals are good models for the study of the cause of the diseases and the analysis of the *Morc2a* gene mechanism. However, it is difficult to obtain mice because of the sublethal characterization shown in our findings. Furthermore, these mice exhibit multiple neuropathies and are not suitable for studying a single characteristic neurological disease.

“[…] we observed neuronal degeneration in our mouse tissues and demonstrated that the cause was apoptosis caused by DNA damage.”

**What has surprised you the most while conducting your research?**

Our initial research goal was to generate a new CMT2Z disease model mouse. We found a CMT disease phenotype of peripheral nerves in our mice. However, since some *MORC2* variants have been reported to cause central neuropathy, we conducted additional experiments in central nervous system tissues. As a result, we found central neuropathy, such as cerebellar atrophy and spinal muscular atrophy found in patients. Furthermore, through pathological studies, we observed neuronal degeneration in our mouse tissues and demonstrated that the cause was apoptosis caused by DNA damage.

**Figure:**
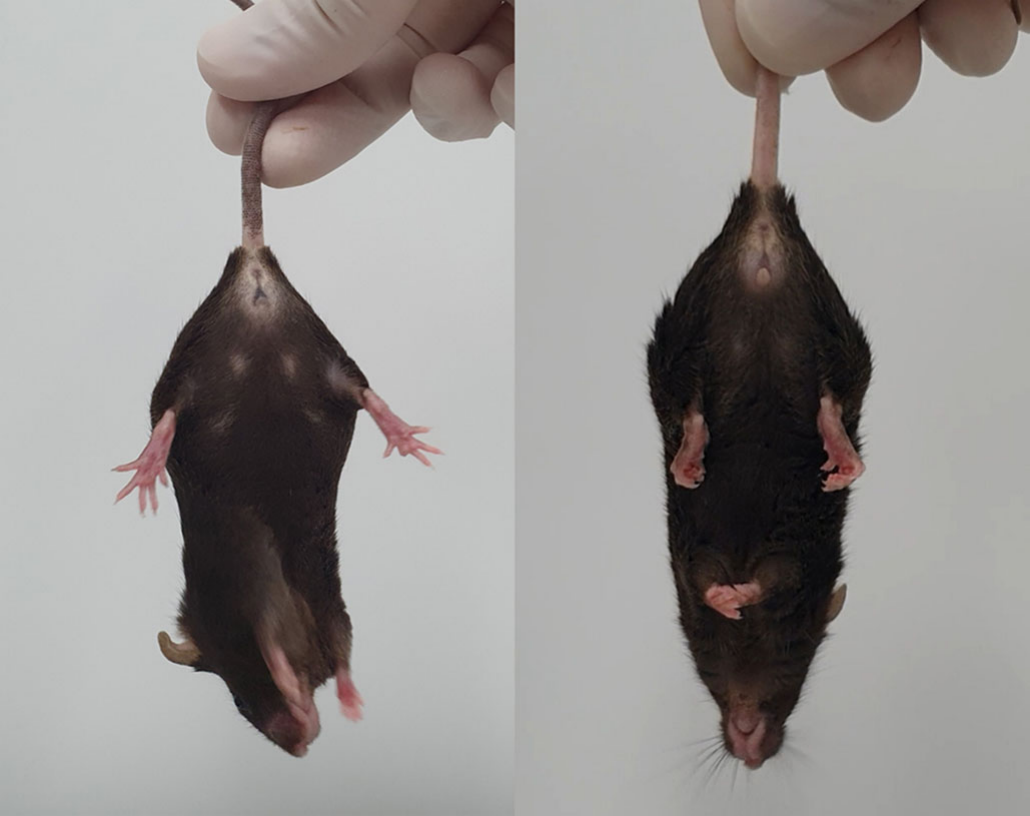
Representative images of wild-type (left) and B6.Morc2a.S87L (right) mice, showing hindlimb clasping.

**Describe what you think is the most significant challenge impacting your research at this time and how will this be addressed over the next 10 years?**

The major challenge in our research is access to therapeutic research. Because our animal model is the first of the *MORC2* variants, it can be used for various studies. However, there are still many things we do not know about the *MORC2* gene, and studies on mechanisms and therapies using this animal must be continued. *Morc2a* S87L/+ mice will be used in preclinical studies for therapy over the next decade. Various drug screenings targeting neuronal degeneration and gene therapy research using CRISPR/Cas9 will be helpful in the treatment of disease.

**What changes do you think could improve the professional lives of early-career scientists?**

In my opinion, I think it is important to exchange with people from other universities or research institutes. We have the opportunity to learn new research from colleagues in various fields. Communication with researchers can encourage further sharing of ideas and setting research goals. This can be a good motivator for early-career scientists.

**What's next for you?**

In terms of this study, the next step is to elucidate the mechanism of disease development. Moreover, gene therapy research with adeno-associated virus is the goal.
